# Sedation for Percutaneous Endoscopic Lumbar Discectomy

**DOI:** 10.1155/2016/8767410

**Published:** 2016-09-22

**Authors:** Menekse Oksar

**Affiliations:** Department of Anesthesiology and Reanimation, Mustafa Kemal University Faculty of Medicine, 31100 Hatay, Turkey

## Abstract

Although anesthetic requirements for minimally invasive neurosurgical techniques have been described in detail and applied successfully since the early 2000s, most of the literature on this subject has dealt with cranial cases that were operated on in the supine or sitting positions. However, spinal surgery has also used minimally invasive techniques that were performed in prone position for more than 30 years to date. Although procedures in both these neurosurgical techniques require the patient to be awake for a certain period of time, the main surgical difference with minimally invasive spinal surgery is that the patients are in the prone position, which may result in increased requirement of airway management because of deep sedation. In addition, although minimally invasive spinal surgery progresses slowly and different techniques are used with no agreement on the terminology used to describe these techniques thus far, the anesthetist needs to understand the surgical and anesthetic requirements for each type of intervention in order to take necessary precautions. This paper reviews the literature on this topic and discusses the anesthetic necessities for percutaneous endoscopic laser surgery.

## 1. Introduction

Endoscopic procedures became widely accepted in the 1990s and substantially contributed to advances in minimally invasive neurosurgery (MIN) in the 2000s [1]. Several percutaneous endoscopic procedures for lumbar disc herniation have been developed since minimally invasive discectomy (MID) was first introduced [1–9]. The central approach for MID shifted toward the posterolateral discectomy approach because of its several advantages, including the fact that it is a more direct approach, prevents nerve injury, and preserves spinal stability [10].

The standard surgical approach for lumbar disc herniation is either open discectomy (OD) or microdiscectomy (MD) [11]. Alternative surgical techniques are available, including MID. The percutaneous endoscopic approach has some advantages over open procedures, such as the preservation of healthy tissue, shorter hospital stays, less blood loss and postoperative pain, and faster patient recovery. However, previous randomized clinical trials on laser discectomy have not provided conclusive evidence on its efficacy because of the small sample sizes [12].

MIN has mostly been used for cranial cases and is performed under sedation [13, 14]. However, optimal sedative and/or sedation protocols for neurosurgical anesthesia have not yet been described [15]. Moreover, there is a lack of knowledge about the optimal position of the patient and their airway management during percutaneous endoscopic laser discectomy (PELD), which are mainly different from MIN procedures in terms of anesthesia. Thus, a comprehensive description of the requirements for MID, particularly for PELD, in terms of sedation/sedoanalgesia and airway management, is required to avoid adverse outcomes and complications during the procedure.

## 2. Evolution of Percutaneous Endoscopic Discectomy Technique and Effectiveness 

PELD has potential advantages over standard surgical procedures for lumbar OD or MD. Percutaneous endoscopy for lumbar discectomy was first described by Kambin and Schaffer in 1973 and percutaneous nucleotomy by Hijikata in 1975, which was referred to as percutaneous discectomy [16, 17], and PELD has been evolving since then because of the need for further development of the technique and equipment. Percutaneous discectomy is a minimally invasive technique that requires a small skin incision and is performed under local anesthesia with or without sedation/sedoanalgesia. However, it is difficult to remove the herniated nucleus pulposus from the channel without an endoscope. Kambin et al. used a posterolateral arthroscopic decompression technique in patients with lateral recess stenosis and reported satisfactory results in 31 of 38 patients (success rate, 82%) [3]. Yeung established the current spinal endoscopic technique in the 1990s, which led to the widespread use of percutaneous discectomy in the 2000s [1].

In addition, Ahn et al. reported the effectiveness of PELD for recurrent disc herniation in selected cases [10], and a systematic review by Rasouli et al. on studies conducted from 1946 to 2013 to compare the pros and cons of MID versus MD/OD revealed contradictory evidence on MID in terms of its potential advantages over MD/OD [11]. Furthermore, in a systematic review of surgical interventions for lumbar disc prolapse, Gibson and Waddell reported that the results of laser discectomy were unclear because only three small randomized clinical trials had been conducted [12]. They also reported that no randomized clinical trials had been conducted on coblation therapy or transforaminal endoscopic discectomy. On the other hand, a multicenter, randomized, open trial on percutaneous laser disc decompression (PLDD) in the Netherlands revealed that PLDD treatment under local anesthesia was noninferior to conventional discectomy performed under spinal or general anesthesia 1 year postoperatively [18].

## 3. Anesthesia for Minimally Invasive Neurosurgery

MIN procedures were performed in the early 2000s in cranial cases, and anesthetic requirements and models have since been described and applied successfully. Percutaneous endoscopic discectomy and MIN for cranial cases have some similarities with regard to their anesthetic and surgical requirements. Awake craniotomy has been performed under sedation; accordingly, the ideal anesthetic for MIN would be administered intravenously, minimally affect brain function, not interfere with electrophysiological monitoring, facilitate neurosurgical procedures, allow the patient's cooperation during surgery, and be associated with rapid and excellent recovery. To date, propofol is the most frequently used agent for this purpose. It enables titratable sedation and rapid and smooth recovery, decreases the incidence of seizures, and when stopped for awakening, minimizes interference with electrocorticographic recordings [15, 19]. Propofol-based anesthesia is still the first-choice hypnotic agent for this purpose, and it has a promising future in this field [19]. In a study comparing combinations of propofol/remifentanil and propofol/sufentanil for supratentorial craniotomy, the former resulted in faster recovery [20]. For lesions close to functional areas, the surgeon may require the cooperation of the patient. In these situations, anesthesia should be such that the patient can cooperate while ensuring adequate depth of anesthesia and airway safety. The technique for performing awake craniotomy is summarized as follows: local anesthesia, sedation, and asleep-awake-asleep technique; airway management has been noted to be the biggest challenge during sedation and transition [21]. Using the same anesthetic combination and infusion technique, Sarang et al. developed an anesthetic technique for awake craniotomy cases, successfully protecting patient safety with adequate acceptability. This technique was a true asleep-awake-asleep technique in which a laryngeal mask airway was used, and remifentanil was administered to produce respiratory depression to control ventilation to a desirable partial pressure of arterial carbon dioxide (PaCO_2_) during the period of being asleep [22]. Moreover, during awake craniotomy, Yamamoto et al. reported successful anesthesia using noninvasive mechanical ventilation (NIMV) with bilevel positive airway pressure in a patient. Proportional assist ventilation was administered to another patient via a nasal mask during tumor resection, with NIMV being discontinued during cortical mapping [21]. Berkenstadt et al. monitored hemodynamic and respiratory status while administering propofol and remifentanil infusion during awake craniotomy and found that overall respiratory complications decreased over the course of the study as the anesthetist gained experience about the study protocol [23]. Keifer et al. reported the timeline of conduction anesthesia with an asleep-awake-asleep technique for awake craniotomy that required brain mapping under propofol and remifentanil infusion; this technique allowed satisfactory anesthetic conditions in most of the patients and a wakeup time of 9 min [24]. The authors also reported that frequent respiratory depression [i.e., PaCO_2_ = 50 (36–69) mmHg, minimum respiratory rate = 0 (0–3) breaths/min, and lowest peripheral capillary oxygen saturation (SpO_2_) = 95% (92–98)] and short hypertensive episodes [maximum value = 150 (139–175) mmHg] were observed during the painful procedures. However, there are important differences between percutaneous endoscopic discectomy and MIN for cranial cases. Deep sedation in the prone position is required during the former, and thus proper management of sedation depth is necessary.

## 4. Sedation Depth during Percutaneous Endoscopic Discectomy 

Besides considering the absolute indications of the sedatives and medications affecting sedation depth, monitoring of sedation can be helpful for the management of PELD cases. The bispectral index score (BIS) can be used to monitor anesthetic depth and is an electroencephalography- (EEG-) derived parameter for the intraoperative monitoring of anesthetic depth [25]; BIS correlates with the level of sedation and loss of consciousness during the administration of propofol, midazolam, isoflurane, or alfentanil [26]. In BIS monitoring of computer-controlled infusion of propofol and remifentanil for awake craniotomy cases, Hans et al. adjusted the target drug concentrations according to the patient's responses to the painful stimuli and to the need for functional/speech testing [27]. They concluded that BIS correlated better with the level of sedation/patient responsiveness than the predicted effect-site concentrations of propofol. Schmidt et al. compared Narcotrend (NT), BIS, and EEG in terms of their ability to monitor the depth of consciousness in different states, from immediately after induction until extubation, in laminectomy cases under propofol and remifentanil anesthesia. The authors found that, for a standardized regimen of propofol and remifentanil, only NT and BIS could accurately distinguish among all the investigated states; remifentanil infusion of 0.3 *µ*g/kg/min during propofol infusion had no statistically significant effects on any of the EEG variables during the 10 min observation period [28]. Sebel et al. examined the correlation between BIS and patients' responses in skin incision study and found that BIS clearly depends on the anesthetic technique and correlates best with the effects of hypnotic-based anesthetic agents. In contrast, opioid-based techniques reduced patients' movements with little effect on EEG confounding the use of BIS for an adequate anesthetic effect [29]. The simultaneous use of different monitoring tools that collect data from different mechanisms may be better than using one tool alone for achieving safe sedation that is not deeper than necessary for the specific surgical intervention. Kasuya et al. investigated the correlations between the BIS value and the Observer's Assessment of Alertness/Sedation (OAA/S) score during separate infusion of propofol and dexmedetomidine in volunteers and found that BIS values were lower for dexmedetomidine at comparable OAA/S scores. They concluded that both scales provide different and complementary data for clinicians and thus suggested that clinicians using dexmedetomidine should use both scales to evaluate a patient's response to sedation [30].

## 5. Suggestions for Handling PELD Cases

The anesthesia technique for MID is chosen depending on the specific procedure, with consideration of whether or not it is an endoscopic intervention. Local anesthesia with conscious sedation is an option in percutaneous endoscopic discectomy procedures, which differ from percutaneous lumbar disc decompression procedures in that the latter are typically performed under local anesthesia. Percutaneous endoscopic discectomy also differs from the standard OD or MD in that the patient can warn the surgeon if the instrumentation impinges on the nerve root during the procedure. However, patients may require extra sedatives or analgesics because of the surgical conditions.

BIS is not affected by noxious stimuli under adequate analgesia; thus, sedation maintenance can be achieved by the concomitant use of analgesics during painful procedures. Appropriate analgesic doses and timing are important for adequately maintaining sedation depth and keeping it within the safe range of sedation levels/scores. Thus, for safe sedation, it is important not to exceed the sedation targets, and different sedation evaluation tools should be used simultaneously rather than one tool alone. In case of respiratory depression, equipment for ventilatory support should be readily available throughout the procedure. Furthermore, conversion from local to general anesthesia should always be an option in prone-positioned patients, which requires complete abandonment of the surgical procedure, followed by endotracheal intubation and repositioning of the patient. Although minimally invasive spinal surgery techniques are continually evolving, anesthetic requirements can be concluded as using sedatives with absolute indications, achieving a safe sedation depth using proper monitoring tools to avoid oversedation, and proper airway management in the prone position.
